# End-of-life decisions in acute stroke patients: an observational cohort study

**DOI:** 10.1186/s12904-016-0113-8

**Published:** 2016-04-05

**Authors:** Angelika Alonso, Anne D. Ebert, Dorothee Dörr, Dieter Buchheidt, Michael G. Hennerici, Kristina Szabo

**Affiliations:** Department of Neurology, UniversitätsMedizin Mannheim, University of Heidelberg, 68167 Mannheim, Germany; Health Care Ethics Committee, UniversitätsMedizin Mannheim, University of Heidelberg, 68167 Mannheim, Germany; Department of Hematology and Oncology, UniversitätsMedizin Mannheim, University of Heidelberg, 68167 Mannheim, Germany

**Keywords:** Stroke, End-of-life decisions, Palliative care, Advance directives, Stroke mortality

## Abstract

**Background:**

Crucial issues of modern stroke care include best practice end-of-life-decision (EOLD)-making procedures and the provision of high-quality palliative care for dying stroke patients.

**Methods:**

We retrospectively analyzed records of those patients who died over a 4-year period (2011–2014) on our Stroke Unit concerning EOLD, focusing on the factors that most probably guided decisions to induce limitation of life-sustaining therapy and subsequently end-of-life-care procedures thereafter.

**Results:**

Of all patients treated at our Stroke Unit, 120 (2.71 %) died. In 101 (86.3 %), a do-not-resuscitate-order (DNRO) was made during early treatment. A decision to withdraw/withhold further life supportive therapy was made in 40 patients (34.2 %) after a mean of 5.0 days (range 0–29). Overall patient death occurred after a mean time of 7.0 days (range 1–30) and 2.6 days after therapy restrictions. Disturbance of consciousness at presentation, dysphagia on day 1 and large supratentorial stroke were possible indicators of decisions to therapeutic withdrawing/withholding. Proceedings of EOL care in these patients were heterogeneous; in most cases monitoring (95 %), medical procedures (90 %), oral medication (88 %), parenteral nutrition (98 %) and antibiotic therapy (86 %) were either not ordered or withdrawn, however IV fluids were continued in all patients.

**Conclusions:**

A high percentage of stroke patients were rated as terminally ill and died in the course of caregiving. Disturbance of consciousness at presentation, dysphagia on day 1 and large supratentorial stroke facilitated decisions to change therapeutic goals thus initiating end-of-life-care. However, there is further need to foster research on this field in order to ameliorate outcome prognostication, to understand the dynamics of EOLD-making procedures and to educate staff to provide high-quality patient-centred palliative care in stroke medicine.

**Electronic supplementary material:**

The online version of this article (doi:10.1186/s12904-016-0113-8) contains supplementary material, which is available to authorized users.

## Background

Despite considerable advances in acute stroke therapy and a decline of the relative rate of stroke deaths [1], stroke remains the third to fourth most common cause of death in the United States and Europe [1–3]. In 2014, the age-adjusted death rate for stroke as an underlying cause of death in the United States was 36.5 per 100,000 [4], with approximately 50 % of stroke deaths occurring in hospital [5]. In 2011, reported admission-based case-fatality rates for ischemic stroke in Germany were 6.7 per 100 discharges, for intracerebral haemorrhage as high as 17.5 per 100 discharges, respectively [2]. In the light of increasing hospitalizations for cerebrovascular diseases [2], palliative care and end-of-life decisions (EOLD) are more and more recognized as a crucial part of modern stroke care. This is even more relevant, as recent data suggest that a substantial proportion of inpatient deaths occur in the context of withdrawal or withholding of life-sustaining therapies in severe stroke patients [6, 7].

EOLD in stroke patients include a broad array of choices ranging from do-not-resuscitate-orders (DNRO) [8, 9], combined with continuance of any other life-supporting measures, to symptom control only and withdrawal or withholding of further therapy [10, 11]. However, the implication of a particular EOLD on the proceedings thereafter remains largely unclear. In a large cohort of stroke patients, a DNRO was associated with an increased risk of death, possibly reflecting less intensive care once a DNRO was made [12]. Moreover, in patients with life-threatening stroke, a palliative care approach might be chosen in order to improve quality of life. To date, there is a broad consensus that there is an enormous but still underestimated need for palliative care in dying stroke patients [13–15]. Whereas end-of-life care is well established in diseases such as cancer or advanced heart disease, research on end-of-life care has been relatively neglected in stroke populations and evaluation of end-of-life care processes in stroke has to date been very limited [16–18]. Moreover, the palliative care needs of acute stroke patients may be very different from those in advanced stages of other chronic diseases [19]. According to a recent scientific statement from the American Heart Association/American Stroke Association, palliative care in stroke should cover complex decision making in terms of uncertainties regarding stroke prognosis and putative patient’s preferences, alignment of treatment with goals and symptom control [13]. Despite elaborate recommendations addressing stroke system care and provider teams, standard operating procedures regarding diagnostic and therapeutic management of dying stroke patients are lacking, possibly due to the complexity of end-of-life issues.

Our study aimed to describe and analyze EOLD of dying stroke patients who died during the in-hospital phase on the Stroke Unit, focusing on possible factors that could indicate the decision for a transition from life-sustaining therapy to withdrawal or withholding of defined diagnostic or therapeutic measures, and the impact of this decision on the proceedings thereafter. Insight from this study will help to raise awareness for palliative care issues in stroke patients and aid the development of recommendations for stroke-specific palliative therapy and EOLD.

## Methods

From our prospectively collected electronic stroke database we retrospectively identified and analyzed the charts of all patients (*n* = 4425) admitted to our 29 bed comprehensive Stroke Unit (SU) with the diagnosis of ischemic stroke or intracerebral hemorrhage (ICH) between January 2011 and December 2014 who died during the course of hospital treatment. Patient data was extracted from the electronic medical records to a specially designed data collection form by two experienced stroke physicians (A.A. and K.S.). In cases of discrepancy, the final interpretation of the data was reached by mutual agreement. In this stroke database, all clinical data and technical investigations were recorded and documented according to a standardized acute stroke care protocol including clinical examination at regular intervals according to standard Stroke Unit requirements in Germany [20]. Among others, all patients also received an assessment of dysphagia on day 1 assessed on the basis of the Dysphagia Outcome and Severity Scale [21] and were classified as either severe, mild to moderate or normal by qualified speech therapists. To avoid any bias, we excluded those 23 patients who were transferred to the intensive care unit prior to death. In these patients, EOLD mostly comprised withdrawal/withholding of intensive care measures such as mechanical ventilation, surgery or circulatory support with catecholamines. These decisions were not in the scope of the current study. However, demographic and baseline data as well as an overview on EOLDs in these patients are available in Additional file 1: Figure S1.

Patient records were further retrospectively analyzed with respect to the ability of the patients to communicate, information about the patient’s wishes concerning end-of-life medical treatments, result of individual EOLD, and medical proceedings thereafter.

Patients were classified according to the ability to communicate as either awake and able to communicate normally, with limited ability to communicate, or not able to communicate due to disturbed consciousness, dys- or aphasia by the treating stroke physician in the context of the neurological examination every 8 h. Patient charts were reviewed for do-not-resuscitate-orders (DNRO) and therapy goal modifications with transition to symptom control care. In this context, the reconstruction of the decision to pursuit a palliative goal was related to the course of action of withholding or withdrawing life-sustaining therapies. The analyzed proceedings following particular EOLD included diagnostic procedures, nutrition, medication and initiation of palliative measures.

The cause of death was classified into neurological (stroke-related/herniation), cardiac (therapy refractory tachy- or bradycardia, acute cardiac insufficiency, acute myocardial infarction), severe infections (pulmonary, abdominal, CNS) and to otherwise specified or undetermined reasons. Multiple assignments were allowed.

In order to analyze possible indicators of transition to palliative care, we performed *χ*^2^-test for categorical and *t*-test for metric variables. The impact of dysphagia, premorbid modified Rankin Scale (mRS) and National Institute of Health Stroke Scale (NIHSS) at admission on the decision for palliative care was calculated by Mann-Whitney-*U*-test. A *p* value < 0.05 was considered to indicate statistical significance. Statistical analysis was performed using the Statistical Package for the Social Sciences (SPSS), version 22.0. (IBM, USA).

## Results

**Fig. 1:**
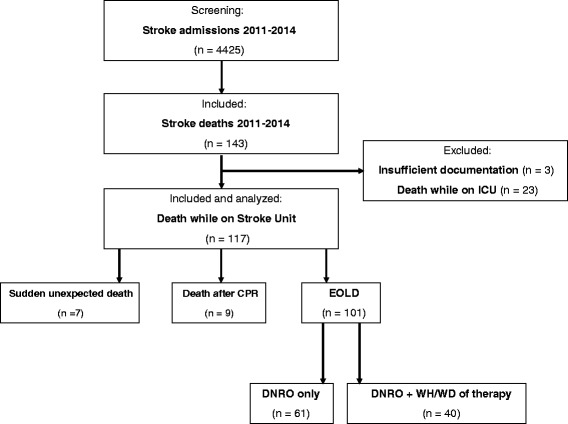
Cohort selection. CPR: cardiopulmonary resuscitation; DNRO: do not resuscitate order; WH/WD: withholding or withdrawal

From January 2011 to December 2014, 4425 patients were admitted to our comprehensive Stroke Unit with the diagnosis of ischemic stroke or intracerebral hemorrhage (ICH). Of these, 143 patients (3.23 %) died during the in-hospital phase; 120 of these deaths (2.71 %) occurred on the Stroke Unit, and 23 on intensive care unit. Three patients had to be excluded due to insufficient documentation, and therefore records of 117 patients (49 male, 68 female, mean age 81.8 years, range 55–96 years, median premorbid mRS 1.5) were finally analyzed in the study (Fig. 1). Initial stroke event occurred due to ischemic stroke in 85 (72.6 %) and intracerebral haemorrhage in 32 patients (27.4 %). Most patients had severe neurological deficits at admission with a mean NIHSS of 19 (range 0–28). Concerning advance directives, 39 patients (33.3 %) had a living will and 45 (38.5 %) had a power of attorney for health care. At the time of admission, 11 patients (9.4 %) had a legal guardian due to premorbid impairments of cognitive, social or other important functions.

In 101 of all analysed patients (86.3 %), a DNRO was made during the early course of treatment, mostly within 48 h after admission when the diagnosis of a life-threatening stroke was established. Nine of the remaining 16 patients died after cardiopulmonary resuscitation failure, the other seven died subsequently to sudden unexpected causes. In none of these patients had EOLDs been discussed. From the 71 patients with a DNRO but no further limitation of therapy, only 2 were able to communicate, 16 had limited ability, and 43 no ability to communicate at the time of EOLD-making. A decision to transition to palliative level of care was made in 40 patients (34.2 %) after a mean time interval of 5.0 days (range 0–29). In all of these 40 cases, a DNRO was already in place at the time of transition. Out of these 40, 2 patients had limited ability to communicate, while the 38 remaining were not able to communicate at the time of decision making. In patients no longer able to communicate, the decision to transition to palliative level of care was either based on an existing living will or in consent with an attorney for health care or legal guardian following the presumed patient’s will. In patients without advance directives or legally designated patient representatives, a consensus about EOLD was found between the patient’s family and the attending neurologists, taking into account the information of the most likely patient’s will provided by the family.

Overall, patient death occurred after a mean time of 7.0 days (range 1–30) from admission. Patients transitioned to palliative care died after a mean of 2.5 days thereafter. Survival time of palliative stroke patients and patients with DNRO or no end-of-life decision did not differ significantly (mean 7.2 days vs. 6.7 days vs. 8.6 days, *p* = 0.494). In 78 cases (66.7 %) death was related directly to stroke. In 13 cases (11.1 %) death was due to severe infection and in 21 cases (17.9 %) due to cardiac disorder, whereas the precise cause of death remained unclear in 9 cases (7.7 %). The baseline characteristics and demographic information of the included patients are given in Table 1.

**Table 1:** Baseline characteristics and demographic information of patients (*n* = 117)

Demographics		
Mean age, years; (range)	81.8	(55–96)
Gender, n female; (%)	68	(58.1)
Median pre-mRS; (range)	1.5	(0–5)
Diagnosis		
Ischemic stroke, n; (%)	85	(72.6)
Intracerebral hemorrhage, n; (%)	32	(27.4)
Stroke severity		
NIHSS at admission, median; (range)	19	(0–28)
Comorbidities		
Dementia, n; (%)	18	(15.4)
Cancer, n; (%)	22	(18.8)
Renal dialysis, n; (%)	3	(2.6)
Referral to other specialties		
Neurosurgeon, n; (%)	15	(12.8)
Otolaryngologist, n; (%)	6	(5.1)
Urologist, n; (%)	6	(5.1)
Oncologist, n; (%)	4	(3.4)
Course		
Days to death, median; (range)	7.0	(1–30)
Death expected, n; (%)	90	(76.9)
Death unexpected, n; (%)	27	(23.1)
Therapy aspects		
DNRO, n; (%)	101	(86.3)
Unsuccessful CPR, n; (%)	9	(7.7)
Transitioned to palliative care, n; (%)	40	(34.2)
Days to transition, mean; (range)	5	(0–29)
Days from transition to death, mean; (range)	2.6	(0–17)
Advance directive		
Living will, n; (%)	39	(33.3)
Power of attorney for health care, n; (%)	45	(38.5)
Legal guardian, n; (%)	11	(9.4)
Cause of death		
Complication of stroke, n; (%)	78	(66.7)
Cardiac disorder, n; (%)	21	(17.9)
Severe infection, n; (%)	13	(11.1)
Other/not known, n; (%)	9	(7.7)

Abbreviations: *pre-mRS* premorbid modified Rankin Scale, *NIHSS* National Institute of Health Stroke Scale, *DNRO* do-not-resuscitate order, *CPR* cardiopulmonary resuscitation

**Table 2:** Possible indicators of transition to palliative care

Variable	Patients transitioned to palliative care (*n* = 40)	Patients not transitioned to palliative care (*n* = 77)	*p*-value
Age (mean, standard deviation)	83.2 +/-8.2y	81.1+/-8.6y	0.194
Diagnosis			
Ischemic stroke	26 (65 %)	59 (76.6 %)	0.132
Intracranial hemorrhage	14 (35 %)	18 (23.4 %)	
Localisation			
supratentorial	35 (87.5 %)	69 (89.6 %)	0.762
infratentorial	5 (12.5 %)	8 (10.4 %)	
Hemisphere			
dominant	14 (35.0 %)	30 (39.0 %)	
non-dominant	17 (42.5 %)	39 (50.6 %)	0.209
bihemispheric	9 (22.5 %)	8 (10.4 %)	
Stroke volume			
Ischemia >2/3 ACM, total ACA or total	22/26 (84.6 %)	33/59 (55.9 %)	**0.009**
ACP territory			
ICH > 60 ml	14/14 (100 %)	14/18 (77.8 %)	0.085
NIHSS at admission			
median (range)	19.5 (4-26)	19 (0-28)	0.115
16 and higher	29 (72.5 %)	51 (66.2 %)	0.317
Item 1a (Level of consciousness) >0	17 (42.5 %)	18 (23.4 %)	**0.028**
Dysphagia assessment on day 1			
Normal	4/33 (12.1 %)	18/70 (25.7 %)	
Mild to moderate	15/33 (45.5 %)	36/70 (51.4 %)	**0.026**
Severe	14/33 (42.4 %)	16/70 (22.9 %)	
Advance directive			
Living will	15 (37.5 %)	24 (31.2 %)	0.538
Power of attorney for health care	20 (50 %)	25 (32.5 %)	0.074
Legal guardian	3 (7.5 %)	8 (10.5 %)	0.747
Pre-mRS (median, range)	2 (0–5)	2 (0–5)	0.578
Dementia	7 (17.5 %)	11 (14.3 %)	0.418

Abbreviations: *ACM* middle cerebral artery, *ACA* anterior cerebral artery, *ACP* posterior cerebral artery, *ICH* intracranial hemorrhage, *NIHSS* National Institute of Health Stroke Scale, *pre-mRS* premorbid modified Rankin Scale.

Significant p-values are illustrated in bold print

Patients transitioned to palliative care with restrictions of further life-prolonging therapy were slightly older than those with DNRO or without treatment limitations, however, age was not significantly associated with the decision for palliative care (*p* = 0.194). The proportion of patients with intracerebral haemorrhage was higher in patients transitioned to palliative care, but failed to reach statistical significance (*p* = 0.132). In patients with ischemic stroke, a large stroke volume (defined as either infarction of > 2/3 of the ACM territory or complete infarction of the ACA or ACP territory) was identified as a possible indicator for initiation of palliative care (*p* = 0.009). In patients with ICH, a large blood volume of > 60 ml was found in all patients transitioned to palliative care, but in only 14/18 without palliative care (*p* = 0.085). Although bihemispheric strokes were more frequent in patients transitioned to palliative care, the affected hemisphere as well as a supra- vs. infratentorial localisation of stroke were equally distributed between the two groups (*p* = 0.762 and 0.209, respectively). Interestingly, NIHSS at admission, being high in most deceased stroke patients, was not different between the two groups and thus no possible indicator for the decision to restrict therapy (*p* = 0.115). Likewise, the proportion of severely affected patients, defined as initial NIHSS of 16 and higher, was not found to be higher in patients with the decision for therapy withdrawal or withholding (*p* = 0.317). However, admission to hospital in a decreased level of consciousness, reflected in > 0 points for Item 1a of the NIHSS score, was clearly associated with transition to palliative care (*p* = 0.028). In 103 patients, an assessment of swallowing, dysphagia and speech could be conducted on day 1. In the remaining, dysphagia assessment on day 1 was not feasible due to decease on the first day or unavailability of speech therapists. Overall, the grade of dysphagia was significantly more severe in those patients with an end-of-life decision therapy restriction (*p* = 0.026). In order to adjust this finding for the initial level of consciousness, the influence of dysphagia was separately analyzed in patients with NIHSS Item1a = 0. The grade of dysphagia remained a possible indicator of the decision for therapeutic limitations even in this collective (*p* = 0.044), indicating that dysphagia should be considered as an independent variable. Existence of advance directives was not associated with a transition to palliative care, although a power of attorney for health care was more frequent in patients under palliative care (*p* = 0.074). Regarding comorbidities, neither the premorbid mRS nor the proportion of patients with previously diagnosed dementia differed significantly between the group of patients transitioned to a palliative level of care and those who were not (*p* = 0.571 and 0.418, respectively; Table 2).

**Fig. 2:**
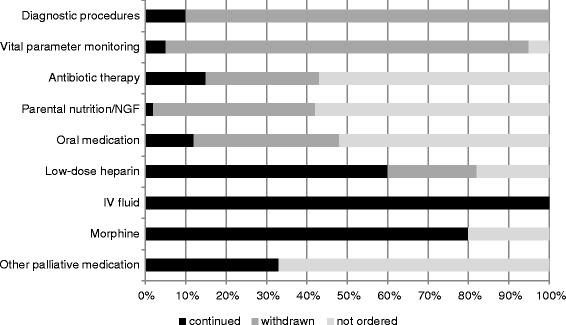
Relative frequencies in percentage of different proceedings after decision to withdraw or withhold therapy (*n* = 40). NGF: Nasogastric feeding; IV: intravenous

After transition to palliative care (*n* = 40), restriction of diagnostic and therapeutic interventions was heterogeneous: Monitoring of vital parameters (95 %) and diagnostic procedures (90 %) were discontinued in most patients. In the palliative care setting, antibiotic therapy (86 %), nutrition (98 %) and oral medication (88 %) were never ordered or withdrawn in the majority of patients. Low-dose heparin was only withdrawn in 9/40 patients. All patients received IV fluids until death, however, mostly adapted to palliative care needs. In the last phase of their lives, 32/40 patients (80 %) were treated with morphine, 13 patients received other palliative medication including opioid and non-opioid analgesics and benzodiazepines (see Fig. 2). Palliative sedation was not performed in any patient due to deep coma in the dying phase in 36/40 patients and good symptom control in the remaining 4/40 patients.

Thirty-five of 61 patients with consented DNRO (57.4 %), received treatment with palliative medication during the last 48 h of life, 25 (40.1 %) of these were on continuous treatment with morphine. Nineteen patients (31.2 %) had additional palliative medication for symptom control, mostly opioid and non-opioid analgesics, antiemetic drugs and benzodiazepines. None of the 16 patients who underwent attempts for cardiopulmonal resuscitation received morphine or other opiods. Two patients were treated with antiemetics, and one patient received neuroleptics due to delirium.

## Discussion

The aim of the present study was to analyze end-of-life stroke care of a cohort of stroke patients who died during the in-hospital phase on the Stroke Unit after suffering an acute ischemic or haemorrhagic stroke. We compared the characteristics of those patients who were transitioned to palliative care as a result of previous EOLD comprising withdrawal/withholding of further treatment options with those who were not. The overall mortality of patients admitted with either ischemic or haemorrhagic stroke was 3.23 %; 2.71 % of all deaths occurred on the Stroke Unit – these patients were included into further analyses. The overall mortality rate is within the range of what can be expected: According to a recent report based on a prospective database of 26 hospitals of the Stroke Register of Northwestern Germany, the observed in-hospital mortality was 4.6 % in 2008 and remained fairly stable in the following years [22]. In 86.3 % of our patients who died during the study period, a DNRO was present at the time of death, and 34.2 % of all patients died after the decision was made to withdraw or withhold life-sustaining therapies. According to cohort studies from the United States and the United Kingdom, the proportion of all ICU deaths – including those in patients with severe strokes - preceded by withdrawal of life support, may range from 0 to 96 % [23, 24]. However, in a recent single centre study, inpatient ischemic stroke deaths occurred almost exclusively after limitations of potentially life-sustaining measures, mostly after withdrawal/withholding of mechanical ventilation or artificial nutrition/hydration [6]. Similar data are available for stroke patients who died during their hospitalization on a Stroke Unit [25]. This and our data indicate that deaths as a consequence of acute stroke are mostly anticipated and occur related to corresponding therapeutic decisions. These numbers also indicate a highly preference-based treatment in severely ill stroke patients [26]. But as recently pointed out, there is further need to better understand the marked variation in the care of these patients and to improve the patient-centeredness of such decisions [27]. In our study, deaths occurred early in the course of disease after a median of 7 days (range 1–30) after stroke onset. This observation is supported by others: Goldacre et al. found that of 7,070 deaths within a year of admission for stroke, 69.4 % were within 30 days, and 91.9 % of these occurred during the initial admission [28]. Those patients who obtained a withdrawal of life support died 2.6 days after this decision was made. This suggests that a change of therapy goal to palliative care occurred at a point at which the prognosis became obvious and death was imminent. A study from Blacquiere et al. reported similar data: Out of 104 patients who died on the Stroke Unit over a 2-year period, 90 % were palliated and died after a median time of 2.6 days after beginning palliative care [25].

Data of our institution indicate a two-stage practice as a result of EOLD making with reference to patients after severe stroke. The first stage includes DNRO with no further escalation of neuro-intensive treatment (including ICU treatment, ventilation) but allowing for potential neurological recovery. The second stage comprises an individually composed withdrawal/withholding of further life-sustaining treatment options. Those patients who were transitioned to the latter form of palliative care were more likely to have a disturbance of consciousness at admission and dysphagia on day 1. They had more frequently suffered an ischemic stroke affecting > 2/3 of the ACM or the complete ACA and PCA territories. However, other individual patient factors such as age, pre-mRS and a previously diagnosed dementia were not different between the two groups. Statistical analysis also prompted no effect of the existence of advance directives on therapy decision. This suggests, that prognostic implications as assessed by the medical team have the highest impact on decision making in these situations. EOLD in stroke patients are highly complex as stroke physicians have to consider both aspects from “evidence-based” medicine and “preference-based” medicine when consulting severely affected stroke patients or their surrogates [29]. Moreover, both evidence-based medicine and preference-based medicine each have their specific uncertainties and pitfalls. Concerning evidence-based medicine, EOLD should be based on an outcome prognosis as precise as possible. Several stroke prognostic models, mostly incorporating initial stroke severity and age amongst other factors, have been proven to be useful in estimating the risk of early stroke mortality and short-term disability [30–32]. However, the definition of an unfavourable outcome is inconsistent among different risk scores. On top of that, preference-sensitive decisions may be biased by the disability paradox [33]: stroke patients may maintain a good quality of life despite severe disability whereas healthy people mostly would rate this outcome as unacceptable. Since guidelines for the treatment of ischemic stroke do not provide specific recommendations related to EOLD, research must focus on improving prognostication and decision-making process and implementing these in standard operating procedures.

Since September 2009, German law regulates that advance directives - if applicable - must be respected in any decision concerning medical treatment [34]. In our series, the prevalence of a living will was low, however similar to reports in other medical specialties [35, 36]. Their presence had no obvious influence on transition to palliative care. This is in line with a recent study from the US: The authors reviewed clinical and radiological records of 28 consecutive patients with a diagnosis of ischemic or haemorrhagic stroke treated in a single stroke centre over a 12-month period. The authors found no differences in most treatment decisions in patient management in the presence of advance health care directives [37]. These findings implicate that advance directives need to be more than a signed form and must include specific documentation [38]. This is especially true for stroke that has its unique illness trajectory with special implications. Unlike in most chronic diseases, severe stroke occurs unexpectedly, patients present at their worst, with an uncertain short-term mortality or a likely long-term disability [39]. And, unlike in other life-threatening disorders, virtually all communication is through surrogate decision makers [27]. Therefore, in order to promote patient-centred end-of-life decision-making, further efforts to reform the expressiveness of advance directives are necessary. This implies active educational programs of the general public and promotion of discussions within families to ensure a best estimation of the patient wishes should it become necessary.

At our institution, any decision made on behalf of these patients – including the details of a decision to withdraw/withhold therapy - is based on an individualized personal agreement between the medical team, the patients and/or their surrogates thus applying the shared decision-making concept. Due to the lack of explicit guidelines for palliative care procedures in stroke patients, our approach comprises likewise partial withdrawal, in which individually selected therapies might be stopped, explaining the heterogeneity of the proceedings. While medical procedures, vital parameter monitoring, oral medication, parenteral nutrition and antibiotic therapy might not be ordered, continued or withdrawn in a high percentage of these patients, our data indicate inconsistent practice beyond the scope of “comfort care only” when it comes to low-dose heparin and IV fluids. However, palliative care in terms of symptom control was also ordered in over half of patients not on a therapy restriction order. This shows that end-of-life care practice is indeed variable, dependent on the ability to manage very dynamic processes, the palliative care qualification of the medical staff and on the institutional resources. In this context, the optimal timing of palliative care measures should not be neglected: A recent AHA/ASA Scientific Statement emphasizes that “palliative care should begin at the diagnosis of an acute, serious, and life-threatening stroke” [13]. Due to the specific illness trajectories in stroke with a median time to death of only 7 days, palliative care can be put on a level with end-of-life care in our cohort. However, it would be interesting to elucidate end-of-life issues in those patients who survived their life-threatening stroke.

One might wonder, why not in more cases at some point an active restriction of therapy was decided, given that all of these patients were severely affected and eventually died. Possible explanations could be fast deterioration or difficulties with the surrogates to accept a change in the focus of care [15]. Could it have been that imminent death was not recognized in some patients? Our documentation is not detailed enough to sufficiently answer these questions. It is obvious that many details remain unclear and that further education and training of the stroke care personnel is essential. Unique characteristics of stroke patients as compared to patients suffering from cancer or chronic diseases comprise a sudden onset of severe functional impairment and possible loss of capacity of decision-making on the patient’s side as well as uncertainties in estimating the prognosis of stroke on the physician’s side. These factors argue for specific guidelines concerning palliative care in stroke patients in order to perceive and meet the needs of severely affected stroke patients.

## Conclusions

In summary, our study shows that in the majority of cases death caused by stroke is expected. In 101 of 117 analysed patients (86.3 %), death occurred either with a DNRO in place or with an additional decision to withdraw or withhold life-sustaining therapy. Patients with therapy restriction had significantly more often a disturbance of consciousness at presentation, dysphagia on day 1 and large supratentorial strokes when compared to those with a DNRO only. After withdrawal or withholding life-sustaining therapy, further proceedings in this cohort indicate individualized decision making but also inconsistencies underlining the need for specific palliative care education and research on this field that guides stroke physicians in their daily practice regarding different aspects in the management of dying stroke patients.

### Ethics approval and consent to participate

The local Institutional Review Board (Medizinische Ethikkommission II der Medizinischen Fakultät Mannheim, University of Heidelberg) approved this cohort study. Approval was obtained to analyse the data from the database. Patient consent was not required due to the retrospective nature of the study and the lack of patient interaction.

### Consent for publication

Not applicable.

## Availability of data and materials

The datasets supporting the conclusions of this article are included within the article and its additional file.

## Additional file

Additional file 1: Figure S1. a. Baseline characteristics and demographic information of patients transferred to Intensive Care Unit (*n* = 22)^1^. b. End-of-life decisions^3^ in dying stroke patients after transferral to Intensive Care Unit (*n* = 22)^1^. (PPT 185 kb)

## Abbreviations

ACA: anterior cerebral artery
ACM: middle cerebral artery
ACP: posterior cerebral artery
AHA/ASA: American Heart Association/American Stroke Association
CPR: cardiopulmonary resuscitation
DNRO: do-not-resuscitate-order
EOLD: end-of-life decisions
ICH: intracerebral hemorrhage
ICU: intensive care unit
mRS: modified Rankin Scale
NIHSS: National Institute of Health Stroke Scale
SU: postroke unit

## References

[1] Go AS, Mozaffarian D, Roger VL, Benjamin EJ, Berry JD, Blaha MJ, Dai S, Ford ES, Fox CS, Franco S (2014). Heart disease and stroke statistics--2014 update: a report from the American Heart Association. Circulation.

[2] Townsend N, Nichols M, Scarborough P, Rayner M (2015). Cardiovascular disease in Europe 2015: epidemiological update. Eur Heart J.

[3] WHO Global Infobase: data for saving lives [https://apps.who.int/infobase/].

[4] Arias E, Kochanek KD, Xu J, Murphy SL. Mortality in the United States,2014. NCHS Data Brief 2015(229):1-8. http://www.cdc.gov/nchs/products/citations.htm. 26727391

[5] Centers for Disease C, Prevention (2006). Place of death after stroke--United States, 1999-2002. MMWR Morb Mortal Wkly Rep.

[6] Kelly AG, Hoskins KD, Holloway RG (2012). Early stroke mortality, patient preferences, and the withdrawal of care bias. Neurology.

[7] Mayer SA, Kossoff SB (1999). Withdrawal of life support in the neurological intensive care unit. Neurology.

[8] Reeves MJ, Myers LJ, Williams LS, Phipps MS, Bravata DM (2012). Do-not-resuscitate orders, quality of care, and outcomes in veterans with acute ischemic stroke. Neurology.

[9] Hemphill JC, Newman J, Zhao S, Johnston SC (2004). Hospital usage of early do-not-resuscitate orders and outcome after intracerebral hemorrhage. Stroke.

[10] Zahuranec DB, Brown DL, Lisabeth LD, Gonzales NR, Longwell PJ, Smith MA, Garcia NM, Morgenstern LB (2007). Early care limitations independently predict mortality after intracerebral hemorrhage. Neurology.

[11] Becker KJ, Baxter AB, Cohen WA, Bybee HM, Tirschwell DL, Newell DW, Winn HR, Longstreth WT (2001). Withdrawal of support in intracerebral hemorrhage may lead to self-fulfilling prophecies. Neurology.

[12] Shepardson LB, Youngner SJ, Speroff T, Rosenthal GE (1999). Increased risk of death in patients with do-not-resuscitate orders. Med Care.

[13] Holloway RG, Arnold RM, Creutzfeldt CJ, Lewis EF, Lutz BJ, McCann RM, Rabinstein AA, Saposnik G, Sheth KN, Zahuranec DB (2014). Palliative and end-of-life care in stroke: a statement for healthcare professionals from the American Heart Association/American Stroke Association. Stroke.

[14] Burton CR, Payne S, Addington-Hall J, Jones A (2010). The palliative care needs of acute stroke patients: a prospective study of hospital admissions. Age Ageing.

[15] Wee B, Adams A, Eva G (2010). Palliative and end-of-life care for people with stroke. Curr Opin Support Palliat Care.

[16] Cowey E, Smith LN, Stott DJ, McAlpine CH, Mead GE, Barber M, Walters M (2015). Impact of a clinical pathway on end-of-life care following stroke: a mixed methods study. Palliat Med.

[17] Gardiner C, Harrison M, Ryan T, Jones A (2013). Provision of palliative and end-of-life care in stroke units: a qualitative study. Palliat Med.

[18] Burton CR, Payne S, Turner M, Bucknall T, Rycroft-Malone J, Tyrrell P, Horne M, Ntambwe LI, Tyson S, Mitchell H (2014). The study protocol of: ‘Initiating end of life care in stroke: clinical decision-making around prognosis’. BMC Palliat Care.

[19] Stevens T, Payne SA, Burton C, Addington-Hall J, Jones A (2007). Palliative care in stroke: a critical review of the literature. Palliat Med.

[20] Weimar C, Ringelstein EB, Diener HC (2007). Monitoring stroke units: management, outcome, efficiency. Nervenarzt.

[21] O’Neil KH, Purdy M, Falk J, Gallo L (1999). The Dysphagia Outcome and Severity Scale. Dysphagia.

[22] Minnerup J, Wersching H, Unrath M, Berger K (2015). Explaining the Decrease of In-Hospital Mortality from Ischemic Stroke. PLoS One.

[23] Prendergast TJ, Claessens MT, Luce JM (1998). A national survey of end-of-life care for critically ill patients. Am J Respir Crit Care Med.

[24] Wunsch H, Harrison DA, Harvey S, Rowan K (2005). End-of-life decisions: a cohort study of the withdrawal of all active treatment in intensive care units in the United Kingdom. Intensive Care Med.

[25] Blacquiere DP, Gubitz GJ, Dupere D, McLeod D, Phillips S (2009). Evaluating an organized palliative care approach in patients with severe stroke. Can J Neurol Sci.

[26] Holloway RG, Tuttle D, Baird T, Skelton WK (2007). The safety of hospital stroke care. Neurology.

[27] Holloway RG, Benesch CG, Burgin WS, Zentner JB (2005). Prognosis and decision making in severe stroke. JAMA.

[28] Goldacre MJ, Roberts SE, Griffith M (2004). Place, time and certified cause of death in people who die after hospital admission for myocardial infarction or stroke. Eur J Public Health.

[29] Quill TE, Holloway RG (2012). Evidence, preferences, recommendations--finding the right balance in patient care. N Engl J Med.

[30] Smith EE, Shobha N, Dai D, Olson DM, Reeves MJ, Saver JL, Hernandez AF, Peterson ED, Fonarow GC, Schwamm LH (2013). A risk score for in-hospital death in patients admitted with ischemic or hemorrhagic stroke. J Am Heart Assoc.

[31] Weimar C, Konig IR, Kraywinkel K, Ziegler A, Diener HC, German Stroke Study C (2004). Age and National Institutes of Health Stroke Scale Score within 6 h after onset are accurate predictors of outcome after cerebral ischemia: development and external validation of prognostic models. Stroke.

[32] Hemphill JC, Bonovich DC, Besmertis L, Manley GT, Johnston SC (2001). The ICH score: a simple, reliable grading scale for intracerebral hemorrhage. Stroke.

[33] Albrecht GL, Devlieger PJ (1999). The disability paradox: high quality of life against all odds. Soc Sci Med.

[34] Wiesing U, Jox RJ, Hessler HJ, Borasio GD (2010). A new law on advance directives in Germany. J Med Ethics.

[35] Graw JA, Spies CD, Wernecke KD, Braun JP (2012). Managing end-of-life decision making in intensive care medicine--a perspective from Charite Hospital, Germany. PLoS One.

[36] Driehorst F, Keller F (2014). Advance directives in patients with kidney disease or other general medical diagnoses. Dtsch Med Wochenschr.

[37] Qureshi AI, Chaudhry SA, Connelly B, Abott E, Janjua T, Kim SH, Miley JT, Rodriguez GJ, Uzun G, Watanabe M (2013). Impact of advanced healthcare directives on treatment decisions by physicians in patients with acute stroke. Crit Care Med.

[38] Suarez JI (2013). Are advance directives useful in acute stroke?*. Crit Care Med.

[39] Mead GE, Cowey E, Murray SA (2013). Life after stroke - is palliative care relevant? A better understanding of illness trajectories after stroke may help clinicians identify patients for a palliative approach to care. Int J Stroke.

