# Supplementary material for: End-of-life decisions in acute stroke patients: an observational cohort study
Source: BMC Palliat Care. 2016 Apr 5;15:38. doi: 10.1186/s12904-016-0113-8 (PMC4820928; doi:10.1186/s12904-016-0113-8)
Supplement: Additional file 1: Figure S1. — a. Baseline characteristics and demographic information of patients transferred to Intensive Care Unit (n = 22)1. b. End-of-life decisions3 in dying stroke patients after transferral to Intensive Care Unit (n = 22)1. (PPT 185 kb) [file 12904_2016_113_MOESM1_ESM.ppt]

## Slide 1
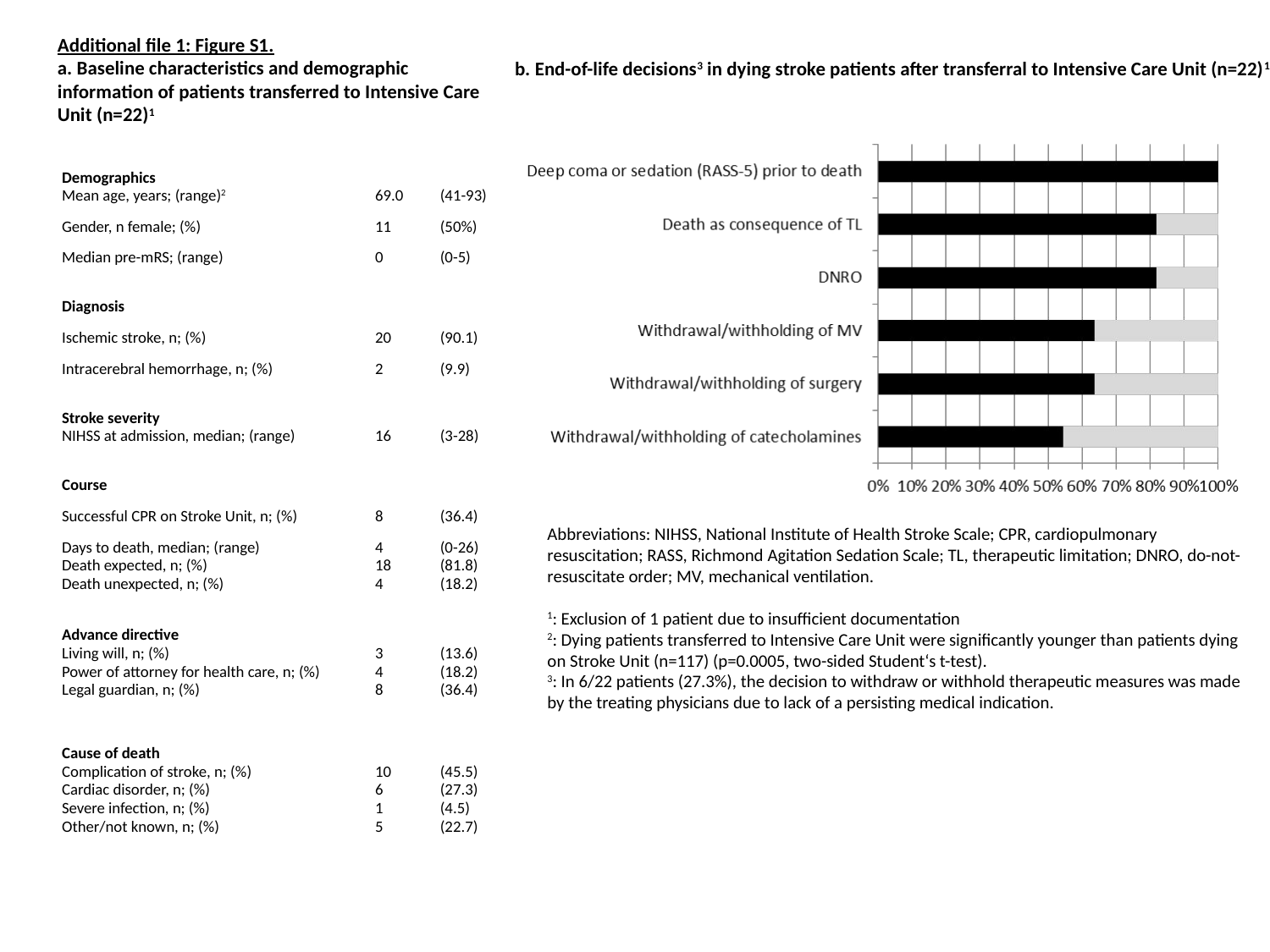

Additional file 1: Figure S1.
a. Baseline characteristics and demographic information of patients transferred to Intensive Care Unit (n=22)1
b. End-of-life decisions3 in dying stroke patients after transferral to Intensive Care Unit (n=22)1
| Demographics Mean age, years; (range)2 | 69.0 | (41-93) |
| --- | --- | --- |
| Gender, n female; (%) | 11 | (50%) |
| Median pre-mRS; (range) | 0 | (0-5) |
| Diagnosis | | |
| Ischemic stroke, n; (%) | 20 | (90.1) |
| Intracerebral hemorrhage, n; (%) | 2 | (9.9) |
| Stroke severity NIHSS at admission, median; (range) | 16 | (3-28) |
| Course | | |
| Successful CPR on Stroke Unit, n; (%) | 8 | (36.4) |
| Days to death, median; (range) Death expected, n; (%) Death unexpected, n; (%) | 4 18 4 | (0-26) (81.8) (18.2) |
| Advance directive Living will, n; (%) Power of attorney for health care, n; (%) Legal guardian, n; (%) | 3 4 8 | (13.6) (18.2) (36.4) |
| | | |
| Cause of death Complication of stroke, n; (%) Cardiac disorder, n; (%) Severe infection, n; (%) Other/not known, n; (%) | 10 6 1 5 | (45.5) (27.3) (4.5) (22.7) |
Abbreviations: NIHSS, National Institute of Health Stroke Scale; CPR, cardiopulmonary resuscitation; RASS, Richmond Agitation Sedation Scale; TL, therapeutic limitation; DNRO, do-not-resuscitate order; MV, mechanical ventilation.
1: Exclusion of 1 patient due to insufficient documentation
2: Dying patients transferred to Intensive Care Unit were significantly younger than patients dying on Stroke Unit (n=117) (p=0.0005, two-sided Student‘s t-test).
3: In 6/22 patients (27.3%), the decision to withdraw or withhold therapeutic measures was made by the treating physicians due to lack of a persisting medical indication.
